# Morphometry of the kangaroo spine and its comparison with human spinal data

**DOI:** 10.1111/joa.13323

**Published:** 2020-10-06

**Authors:** Hans‐Joachim Wilke, Volker Michael Betz, Annette Kienle

**Affiliations:** ^1^ Institute of Orthopaedic Research and Biomechanics Trauma Research Centre Ulm University of Ulm Ulm Germany; ^2^ RKU – University and Rehabilitation Clinics Ulm Ulm Germany; ^3^ SpineServ GmbH & Co. KG Ulm Germany

**Keywords:** animal model, biomechanics, kangaroo, morphometry, spine

## Abstract

The upright posture of the kangaroo suggests that the spine of the kangaroo could be a possible substitute model for biomechanical studies of the human spine. A prerequisite for this should be the agreement of anatomy in humans and kangaroos. The purpose of this study was to investigate the anatomical parameters of the kangaroo spine from C4 to S4 and compare them with existing anatomical data of the human spine. Eight complete spines of the red giant kangaroo were obtained and 21 anatomical parameters were measured from the vertebral bodies, spinal canal, endplate, pedicles, intervertebral discs, transverse, and spinous processes. Most similarities between kangaroo and human spines were found for the vertebral bodies in the cervical and the lumbar spine. The largest differences were evident for the spinous processes. Although both species are somehow upright, these differences may be explained by the way how they move. Jumping probably requires more muscle strength than walking on two legs.

## Graphical abstract

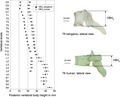

## 1 INTRODUCTION

The best test objects in spine research for biomechanical in vitro investigations are fresh human specimens (Wilke et al., 1998).

Unfortunately, the availability of human spine specimens is limited and expensive. Due to the difficulties of obtaining healthy human spines, the test groups are often inhomogeneous (Ashman et al., 1989); furthermore, spinal specimens of humans hold the risk of infection with hepatitis or AIDS (Cavanaugh & King, 1990). Animal spines are a potential surrogate for *in vitro* testing and have the advantages of better homogeneity, ready availability, and lower risk of infection.

However, in order to be able to use an animal model as a surrogate for human specimens, further conditions must be fulfilled. The biomechanical characteristic of motion segments and the bone density of the vertebral bodies, as well as the anatomy of the vertebral structures and intervertebral discs should be largely similar.

Large quadrupeds are popular models for mechanical testing and numerous studies have characterized their properties. Wilke et al. compared the anatomy of the whole sheep spine and Kandziora et al. the cervical region also from the sheep spine with human data. (Kandziora et al., 2001; Wilke et al., 1997). Other authors investigated and compared the anatomy of different regions of the porcine spine (Bozkus et al., 2005; Dath et al., 2007; Miranpuri et al., 2018; Sheng et al., 2016; Yingling et al., 1999). Kumar et al. performed similar anatomical comparisons for deer spine in the thoracic and lumbar region (Kumar et al., 2000), and Cotterill et al. and Sheng et al. characterized parts of the calf anatomy (Cotterill et al., 1986; Sheng et al., 2016).

The red giant kangaroo (*Macropus rufus*) and the grey kangaroo (*Macropus giganteus*) belong to the species of kangaroos that are comparable in size and weight with humans (Dawson, 1995). This Australian marsupial moves on two legs and often takes an upright posture. Therefore, it might be speculated that there could be some similarities to the human spine. The population in Australia is large and the animals do not belong to a protected species, therefore, they may be available for testing. Boszczyk et al. examined the functional anatomy of the vertebrae in the lumbar region of the grey kangaroo and other mammalian species (Boszczyk et al., 2001), Balasubramanian et al. recently evaluated the thoracic spine morphology of the grey kangaroo (Balasubramanian et al., 2016), and morphological characteristics of the grey kangaroo lumbar intervertebral disc were investigated by Chamoli et al. (2019).

To our knowledge, until now, comprehensive quantitative anatomical data of the whole kangaroo spine are not available in the literature.

The aim of the present study was to determine anatomical parameters from single vertebra of the spine of the red giant kangaroo (*Macropus rufus*) and compare them with existing anatomical data of the human spine.

## 2 MATERIALS AND METHODS

**Figure 1:**
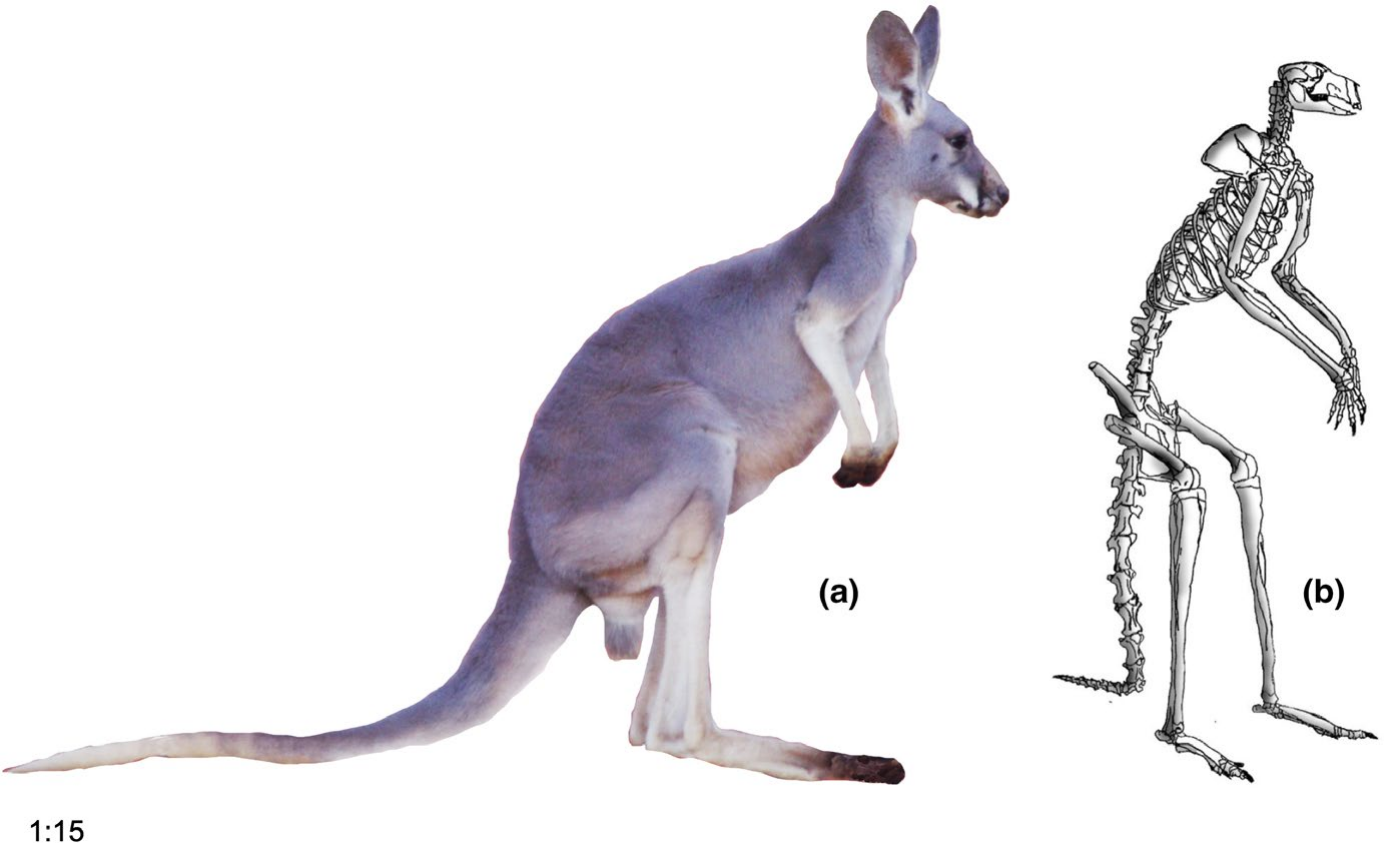
The red kangaroo (*Macropus rufus*) in nature (a) and its skeleton (b)

The anatomical measurements were carried out on n = 8 red giant kangaroos (*Macropus rufus*) (Figure 1). During standing, this kangaroo type can reach a height of 1.8 m. Males usually weigh around 55 kg (max. 90 kg) and females around 23–30 kg (max. 40 kg) (Dawson, 1995).

**Figure 2:**
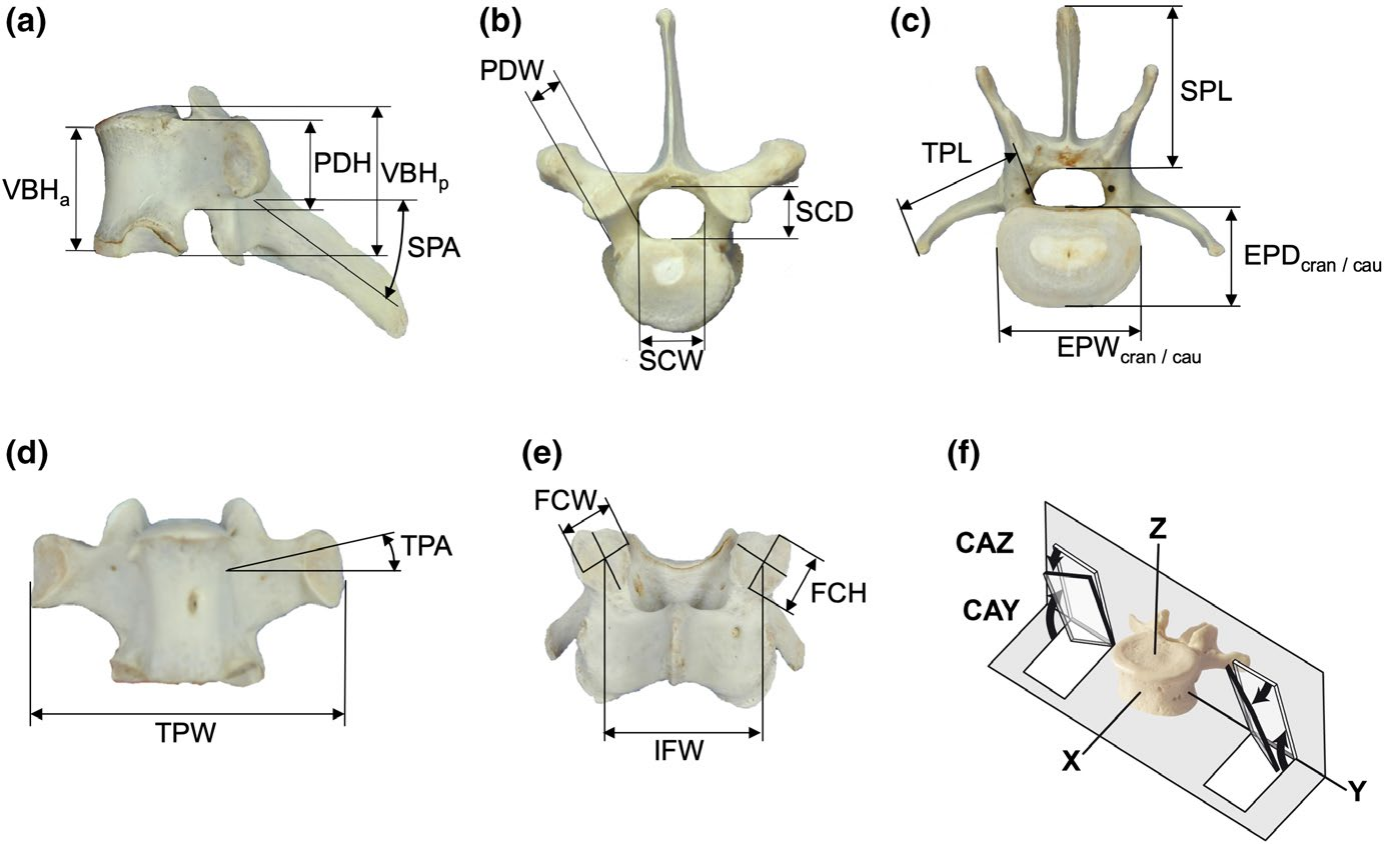
Anatomical definition of reported dimensions on the kangaroo spine. Abbreviations are listed in Table 1. (a) T6, lateral view. (b) T6, cranial view. (c) L4, cranial view. (d) T6, ventral view. (e) C4, dorsal view. (f) Thoracic human vertebra, oblique perspective (adapted from Panjabi et al., 1993)

A kangaroo spine is composed of 7 cervical, 13 thoracic, 6 lumbar, 2 sacral, and 15–20 coccygeal vertebrae. The entire spinal column was removed directly after slaughter and stored at ‐ 20° C until testing. Before preparation the spines were thawed at room temperature. All muscles were dissected and ligaments and intervertebral discs were kept intact in order to maintain the physiological position of the spinal column. After measuring the anterior disc height, the ligaments were removed and the intervertebral discs bisected. Twenty‐one parameters of vertebral dimensions were determined (Table 1, Figure 2) from the dissected vertebral bodies. Because of a special slaughtering procedure, the number of cervical vertebrae was heterogeneous; for this reason it was only possible to measure reliably from C4. From C4‐S4, n = 8 individual values were determined for each measured parameter and mean and standard deviation were calculated.

**Table 1:** Anatomical measuring parameters and the corresponding abbreviations

Vertebral part	Abbreviation	Declaration
Vertebral body	VBH_a/p_	Vertebral body height anterior/posterior
	EPW_cran/cau_	End‐plate width cranial/caudal
	EPD_cran/cau_	End‐plate depth cranial/caudal
Pedicle	PDH	Pedicle height
	PDW	Pedicle width
Spinal canal	SCW	Spinal canal width
	SCD	Spinal canal depth
Spinous process	SPL	Spinous process length
	SPA	Spinous process angle
Transverse process	TPW	Transverse process width
	TPL	Transverse process length
	TPA	Transverse process angle
Articular facet	FCH	Facet height
	FCW	Facet width
	IFW	Interfacet width
	CAY^a^	Card angle about y‐axis
	CAZ^a^	Card angle about z‐axis
Intervertebral disc	IDH_a_	Intervertebral disc height anterior

^a^ The two angles CAY and CAZ represent tilting angles of a card, which were tilted starting from the transversal plane first around the y‐axis (CAY) and then around the z‐axis (CAZ), in order ultimately to lie in the corresponding joint surface planes.

### 2.1 Linear dimensions

All heights, widths, and lengths were measured using sliding calipers (Figure 2). Because of the vertebral symmetry, the pedicle height and width as well as the transverse process length were measured only on the right side. The inter‐joint surface width was measured cranially and the joint surface height and width cranially on the right. Accuracy of measurements was determined by the definition of the anatomical landmarks, the intra‐rater reliability with two measurements per parameter of these results was about 0.5 mm.

### 2.2 Angular dimensions

Angles were measured with a three‐dimensional goniometric linkage system with six rotatory potentiometers with a verified accuracy of 0.1° and 0.1 mm. (Figure 2) (Wilke et al., 1994). Because of the symmetry, the transverse process angle was determined only on the right side, and the articular facet surface inclinations only on the right cranial plane based on the paper of Panjabi et al. (1993).

The cranial endplate represents the transversal reference plane. The middle of the vertebral body and the top of the spinous process represent the sagittal reference plane and the frontal reference plane intersects the cranial endplate orthogonally passing through the tips of the transverse processes.

## 3 RESULTS

### 3.1 Vertebral bodies

The anterior vertebral body height (VBHa) increased from about 20 mm in the cervical region (C5) to about 40 mm in the lumbar spine and in the sacrum (Table 2). In the thoracic region the VBHa increased steadily from 23.5 mm to 28.0 mm and in the lumbar region continues to rise from 30.6 mm to 38.0 mm. In the sacral region from S1 to S3, the mean values of VBHa were constant around 36.4 mm. However, the VBHa of S4 was the highest with 43.5 mm.

**Table 2:** Dimensions relating to the vertebral body of the kangaroo (n = 8; mean ±SD in mm).

Vertebra	VBH_a_	VBH_p_	EPW_cran_	EPW_cau_	EPD_cran_	EPD_cau_
C4	22.0 ± 1.0	22.0 ± 1.0	20.0 ± 0.0	17.3 ± 0.6	11.0 ± 1.0	13.3 ± 1.5
C5	20.6 ± 1.3	20.9 ± 1.4	20.1 ± 1.1	15.9 ± 1.9	10.6 ± 0.5	12.0 ± 1.3
C6	21.0 ± 0.9	20.9 ± 1.1	20.0 ± 1.1	16.8 ± 1.2	10.9 ± 0.4	12.1 ± 0.8
C7	21.6 ± 0.9	21.3 ± 1.8	20.4 ± 0.9	17.8 ± 1.5	11.1 ± 0.6	13.3 ± 1.0
T1	23.5 ± 2.3	22.8 ± 1.8	21.6 ± 1.4	22.9 ± 1.7	12.0 ± 1.1	13.9 ± 1.3
T2	23.9 ± 2.1	23.1 ± 1.6	16.4 ± 0.9	22.3 ± 2.3	12.1 ± 1.0	13.9 ± 1.3
T3	24.4 ± 1.9	23.9 ± 1.1	16.0 ± 0.8	22.0 ± 2.1	12.4 ± 0.5	13.5 ± 1.2
T4	24.8 ± 1.5	24.6 ± 1.3	16.5 ± 0.5	21.1 ± 1.6	13.0 ± 0.5	14.0 ± 1.2
T5	25.4 ± 1.3	25.3 ± 1.3	16.4 ± 0.5	19.3 ± 1.4	13.6 ± 0.7	14.9 ± 1.1
T6	25.3 ± 1.6	25.6 ± 1.7	16.5 ± 0.8	19.4 ± 1.8	14.8 ± 0.7	15.4 ± 0.9
T7	25.5 ± 1.3	25.8 ± 1.8	16.6 ± 0.7	19.5 ± 2.3	15.6 ± 0.7	16.3 ± 1.0
T8	26.3 ± 1.0	26.4 ± 0.9	16.3 ± 0.5	17.6 ± 2.7	15.6 ± 0.9	16.5 ± 1.1
T9	26.4 ± 1.3	26.8 ± 1.0	16.1 ± 0.6	19.4 ± 3.6	15.8 ± 0.9	17.3 ± 1.0
T10	26.6 ± 1.1	27.1 ± 1.1	16.5 ± 0.5	21.1 ± 3.5	16.6 ± 0.7	18.3 ± 1.0
T11	26.8 ± 0.9	27.5 ± 0.8	17.9 ± 1.1	23.4 ± 3.2	17.5 ± 0.9	18.8 ± 1.3
T12	27.3 ± 0.7	28.3 ± 0.9	18.9 ± 1.3	25.6 ± 2.7	18.1 ± 1.1	19.4 ± 1.1
T13	28.0 ± 0.9	29.4 ± 1.3	21.0 ± 1.1	27.3 ± 2.6	18.8 ± 1.0	21.0 ± 1.6
L1	30.6 ± 1.7	32.3 ± 2.7	26.3 ± 1.8	28.6 ± 1.4	21.8 ± 1.6	23.3 ± 2.1
L2	33.5 ± 2.6	34.6 ± 3.0	28.0 ± 1.3	31.6 ± 1.9	23.0 ± 1.3	24.9 ± 2.3
L3	36.9 ± 2.3	38.8 ± 3.4	30.6 ± 0.9	34.3 ± 2.7	25.4 ± 1.5	26.9 ± 1.9
L4	38.1 ± 2.1	40.0 ± 2.6	34.3 ± 1.2	36.6 ± 2.6	26.4 ± 2.0	27.0 ± 2.1
L5	38.4 ± 2.0	40.9 ± 2.3	38.0 ± 1.9	43.1 ± 3.5	26.1 ± 2.4	27.1 ± 1.9
L6	38.0 ± 2.5	39.6 ± 2.8	40.6 ± 3.5	45.0 ± 4.1	25.9 ± 1.9	27.8 ± 1.7
S1	36.5 ± 2.9	37.6 ± 3.1	31.4 ± 1.9	32.4 ± 2.4	25.5 ± 1.9	26.0 ± 2.0
S2	36.5 ± 2.5	38.0 ± 3.4	31.5 ± 2.1	31.9 ± 2.8	25.5 ± 1.9	25.4 ± 2.3
S3	36.2 ± 0.8	37.8 ± 0.8	30.8 ± 1.0	30.5 ± 2.1	25.3 ± 0.8	25.5 ± 1.6
S4	43.8 ± 2.5	44.5 ± 2.6	30.3 ± 1.0	29.8 ± 1.5	25.3 ± 0.8	25.8 ± 0.8

The course of posterior vertebral body height (VBHp) along the spinal column corresponded to its anterior VBHa. Comparing the values of both heights directly, no trend was observed in the cervical region. However, in the upper thoracic region, the VBHp was smaller and caudally from T6 the vertebrae had anterior wedging with VBHp larger than VBHa.

The width of the cranial endplate (EPWcran) was around 20 mm in the cervical region. In the thoracic region, the values ranged between 16.0 and 21.0 mm, in which the middle thoracic region from T2 to T10 was very constant with around 16 mm. The EPWcran in the lumbar region increased steadily from 26.3 mm to 40.6 mm and in the sacral region EPWcran decreased again and was around 31 mm. The caudal endplate width (EPWcau) was in the cervical region between 15.9 and 17.8 mm smaller than the values of EPWcran. However, EPWcau started in the thoracic region with 22.9 mm and decreased steadily to 17.6 mm (T8) and increased again continuously until 45.0 mm in L6. In the sacral region, the EPW decreased again and was nearly the same for both, cranial and caudal endplates.

Depth of the cranial (EPDcran) and caudal (EPDcau) endplates was similar, and both increased in craniocaudal direction. The values in the cervical region were around 11 mm, in the thoracic and lumbar region they increased from ca. 12 to 27 mm, and remained almost the same in the sacral region.

### 3.2 Pedicles

The pedicle height (PDH) in the cervical spine is between 11.8 mm (C6) and 16 mm (C4) (Table 3).

**Table 3:** Dimensions relating to the pedicles and the spinal canal of the kangaroo (n = 8; mean ±SD in mm)

Vertebra	PDH	PDW	SCW	SCD
C4	16.0 ± 1.0	6.7 ± 0.6	14.0 ± 1.0	9.7 ± 0.6
C5	13.1 ± 2.1	6.4 ± 1.1	13.7 ± 0.5	9.7 ± 1.1
C6	11.8 ± 1.7	6.9 ± 0.8	14.1 ± 0.6	10.6 ± 1.9
C7	12.3 ± 1.3	7.1 ± 0.6	14.5 ± 0.8	11.1 ± 1.6
T1	12.5 ± 0.5	8.6 ± 1.4	14.9 ± 2.2	10.3 ± 1.7
T2	14.3 ± 0.7	7.1 ± 0.4	12.0 ± 1.2	8.8 ± 0.9
T3	16.1 ± 2.3	6.5 ± 0.5	11.8 ± 1.0	8.6 ± 0.7
T4	17.9 ± 3.0	5.9 ± 0.4	12.1 ± 1.0	8.3 ± 0.7
T5	17.8 ± 2.5	5.8 ± 0.5	11.9 ± 0.8	8.0 ± 0.9
T6	17.9 ± 2.4	5.6 ± 0.9	11.0 ± 0.5	7.5 ± 0.8
T7	18.8 ± 1.5	5.6 ± 0.7	10.4 ± 0.5	7.6 ± 0.7
T8	17.8 ± 1.2	5.9 ± 0.8	10.8 ± 0.7	7.5 ± 0.8
T9	18.5 ± 0.8	5.9 ± 0.6	11.6 ± 0.7	8.1 ± 0.6
T10	20.1 ± 1.4	7.3 ± 0.9	11.8 ± 0.5	8.5 ± 1.1
T11	20.3 ± 1.3	7.6 ± 1.1	12.8 ± 1.0	8.4 ± 1.1
T12	20.1 ± 1.3	8.0 ± 1.7	12.9 ± 1.0	8.9 ± 1.1
T13	21.8 ± 1.3	8.5 ± 1.7	13.4 ± 1.1	9.1 ± 0.8
L1	25.1 ± 1.6	7.4 ± 1.9	14.3 ± 1.3	9.9 ± 0.6
L2	28.1 ± 2.4	7.1 ± 1.7	14.4 ± 0.9	10.0 ± 0.5
L3	28.4 ± 2.5	7.3 ± 2.0	15.6 ± 1.5	10.3 ± 0.7
L4	29.4 ± 1.5	7.4 ± 1.9	17.0 ± 2.1	10.8 ± 0.5
L5	27.4 ± 2.5	9.0 ± 2.6	18.9 ± 1.7	11.0 ± 0.5
L6	24.0 ± 2.1	9.9 ± 1.9	20.8 ± 1.8	10.6 ± 0.5
S1	28.5 ± 2.1	7.5 ± 0.9	14.9 ± 2.0	6.6 ± 1.2
S2	28.3 ± 2.1	6.9 ± 0.8	15.3 ± 2.1	6.3 ± 0.5
S3	27.3 ± 1.2	6.3 ± 0.5	13.5 ± 0.6	5.8 ± 0.8
S4	33.2 ± 1.6	6.0 ± 0.8	12.5 ± 1.1	1.8 ± 0.8

From the thoracic section there is an almost continuous increase in the measured values, starting with 12.5 mm in T1 up to 33.2 mm in S4.

Pedicle width (PDW) varied between 5.6 mm in T6/T7 and 9.9 mm in L6.

### 3.3 Spinal canal

Spinal canal width (SCW) showed in the thoracic region from T2 till T12 and in S4 lower mean values than in the other regions, in the mid‐thoracic spine (T7), the value was narrowest with 10.4 mm (Table 3). The widest SCW was measured at L6 with a mean of 20.8 mm.

The spinal canal depth (SCD) was similar to the SCW, but always smaller. However, the minimum SCD was found in S4 with 1.8 mm and the largest value was found in C7 with 11.1 mm.

The transversely oval form of the spinal canal was most pronounced in the lumbar and sacral region.

### 3.4 Spinous and transverse processes

Spinous process length (SPL) was lowest in the cervical region with a range from 14.7 mm at C4 to 23.3 mm at C7 (Table 4). The transition to the thoracic region was characterized by a step to 51.3 mm at T1. A maximum was reached at T2 with a mean of 58.6 mm and then decreased to 29.1 mm at T12. A relatively constant SPL were found for lumbar spinous processes between 37.5 mm to 45.1 mm. In the sacral region, the SPL decreased again to 19.0 mm at S3.

**Table 4:** Dimensions and orientation of the spinous and transverse processes of the kangaroo (n = 8; mean ± SD in mm and °)

Vertebra	SPL mm	SPA °	TPW mm	TPL mm	TPA °
C4	14.7 ± 1.5		50.7 ± 5.5	28.3 ± 1.2	49.3 ± 4.5
C5	14.9 ± 0.7		51.6 ± 4.1	24.9 ± 1.4	48.3 ± 6.2
C6	18.9 ± 2.4		54.6 ± 4.8	24.3 ± 1.3	20.5 ± 10.9
C7	23.3 ± 2.2		56.8 ± 3.9	24.3 ± 2.0	15.3 ± 4.4
T1	51.3 ± 2.1	5.8 ± 4.0	63.0 ± 3.9	25.3 ± 2.1	8.3 ± 1.5
T2	58.6 ± 3.6	22.5 ± 3.2	57.0 ± 2.6	22.5 ± 1.6	−19.4 ± 3.0
T3	57.4 ± 6.4	34.1 ± 4.4	53.0 ± 1.5	21.9 ± 1.0	−21.0 ± 2.6
T4	55.5 ± 7.1	40.0 ± 4.5	50.4 ± 1.2	21.1 ± 1.1	−13.9 ± 5.0
T5	54.1 ± 6.5	45.5 ± 2.1	46.0 ± 2.6	20.9 ± 2.5	−2.5 ± 3.8
T6	50.3 ± 7.7	39.4 ± 2.7	43.3 ± 2.7	20.5 ± 2.3	−1.4 ± 2.6
T7	48.0 ± 5.7	37.9 ± 3.8	42.0 ± 4.1	19.1 ± 1.6	3.6 ± 2.8
T8	41.0 ± 3.6	29.9 ± 2.7	40.3 ± 3.7	19.5 ± 1.8	7.1 ± 2.5
T9	35.9 ± 4.5	20.9 ± 5.3	39.1 ± 3.4	18.9 ± 1.9	8.4 ± 1.9
T10	30.3 ± 3.8	5.4 ± 4.0	41.5 ± 2.9	18.9 ± 2.5	17.8 ± 3.2
T11	29.5 ± 4.3	−10.6 ± 2.1	45.6 ± 4.2	20.4 ± 1.9	24.9 ± 4.1
T12	29.1 ± 3.2	−14.0 ± 2.6	49.4 ± 3.9	22.0 ± 1.8	
T13	30.4 ± 2.3	−10.0 ± 2.4	48.0 ± 5.9	20.6 ± 1.6	
L1	37.5 ± 1.6	−20.4 ± 2.8	42.0 ± 6.4	24.1 ± 3.4	
L2	45.1 ± 4.3	−14.1 ± 1.4	41.9 ± 2.3	22.8 ± 1.9	−11.0 ± 2.6
L3	43.9 ± 2.3	−12.0 ± 3.3	46.8 ± 2.7	25.3 ± 0.7	−10.5 ± 1.9
L4	43.4 ± 3.1	−8.5 ± 2.5	58.5 ± 3.8	28.5 ± 1.3	−1.9 ± 3.5
L5	42.4 ± 6.1	−3.3 ± 7.5	67.0 ± 4.6	28.5 ± 2.3	8.6 ± 3.1
L6	38.6 ± 3.6	−11.6 ± 3.5	74.6 ± 3.6	28.9 ± 2.0	10.3 ± 2.1
S1	27.1 ± 3.6		65.1 ± 7.1	50.9 ± 5.0	
S2	26.4 ± 4.9		70.0 ± 2.2	55.3 ± 9.0	
S3	19.0 ± 2.1		68.3 ± 2.6	61.0 ± 3.7	
S4	19.7 ± 4.8		66.4 ± 5.8	60.3 ± 2.7	

The minimum of transverse process length (TPL) was measured in the thoracic region with 18.9 mm at T9‐T10 and the maximum in the lower sacral region with 61.0 mm at S3. The cervical and lumbar regions had similar TPL between 20 and 30 mm. The transverse process width (TPW) was lowest in the thoracic region of T9 with 39.1 mm and the maximum value up to 74.6 mm was measured in L6. The whole sacral region had relatively high values between 65.1 mm and 70.0 mm.

Because of less SPL in the cervical and sacral regions the spinous process angle (SPA) could not be detected in these regions. From T1 SPA tilted caudally by 5.8° and to T5, the angle increased till 45.5°. From T6 the spinous process reappeared in cranial direction and from T11 the spinous process tilted cranially by −10.6°. In the further craniocaudal course, not much changed in the cranial orientation.

The transverse processes were tilting in the cervical region caudally with a transverse process angle (TPA) of 49.3° at C4 to 15.3° at C7. From T2 to T6 the TPA reappeared and tilted cranially from −21.0° to −1.4°. From T7 the transverse processes bent again caudally till 24.9° at T11. Because of the low TPL from T12 to L1, TPA could not be determined in this region. In the lumbar region, TPA inclined between −11.0° and 10.3°.

#### 3.4.1 Facet joints

The smallest articular facet surfaced with the smallest facet heights (FCH) and facet width (FCW) was found in the middle thoracic region with little values from 9.0 mm in T8 for FCH and 6.9 mm in T9 for FCW (Table 5). In the other regions, the articular facet surfaces were larger and ranged between 13.3 and 15.9 mm for FCH and between 10.0 and 16.0 mm for FCW.

**Table 5:** Dimensions and orientation of the cranial articular facet surfaces (n = 8; mean ± SD in mm and °)

Vertebra	FCH mm	FCW mm	IFW mm	CAY °	CAZ °
C4	15.3 ± 2.5	12.3 ± 1.5	25.3 ± 0.6	−62.7 ± 1.5	−23.0 ± 5.6
C5	13.9 ± 1.4	11.9 ± 1.4	26.0 ± 1.4	−64.3 ± 3.7	−30.4 ± 4.6
C6	14.0 ± 2.1	12.3 ± 1.4	25.9 ± 1.3	−65.9 ± 4.0	−31.5 ± 6.7
C7	14.3 ± 1.8	11.9 ± 1.0	25.5 ± 0.8	−69.4 ± 2.6	−38.4 ± 4.5
T1	14.6 ± 1.4	11.8 ± 0.7	24.9 ± 0.6	−72.1 ± 3.4	−41.8 ± 4.5
T2	14.1 ± 2.1	11.0 ± 1.4	22.9 ± 0.6	−61.0 ± 3.1	39.0 ± 4.4
T3	12.3 ± 0.9	10.0 ± 1.1	21.3 ± 1.2	−64.8 ± 2.3	36.4 ± 2.0
T4	11.1 ± 0.6	8.3 ± 0.5	19.6 ± 0.5	−70.8 ± 4.1	42.3 ± 3.3
T5	10.5 ± 0.9	8.0 ± 0.5	18.8 ± 0.7	−70.3 ± 1.9	36.0 ± 4.6
T6	9.8 ± 0.5	7.8 ± 0.5	18.0 ± 0.5	−61.5 ± 4.8	34.0 ± 2.1
T7	9.4 ± 0.7	7.6 ± 0.5	17.8 ± 0.5	−58.3 ± 4.4	36.6 ± 3.9
T8	9.0 ± 0.8	7.1 ± 0.4	17.4 ± 0.5	−61.6 ± 2.5	36.8 ± 3.4
T9	9.1 ± 1.0	6.9 ± 0.8	17.8 ± 0.7	−62.8 ± 3.9	39.8 ± 3.0
T10	9.6 ± 1.2	7.8 ± 1.2	18.1 ± 0.6	−65.5 ± 5.2	38.6 ± 2.7
T11	10.6 ± 1.2	8.4 ± 0.9	18.3 ± 0.5	−53.5 ± 11.4	42.0 ± 2.6
T12	12.4 ± 2.3	10.1 ± 2.0	18.3 ± 1.0	−75.5 ± 9.3	−49.1 ± 14.9
T13	13.6 ± 1.2	12.5 ± 1.8	20.3 ± 1.7	−84.8 ± 2.3	−59.5 ± 3.5
L1	14.4 ± 2.1	15.1 ± 2.1	22.6 ± 1.1	−85.9 ± 3.4	−53.8 ± 4.2
L2	15.9 ± 1.7	16.0 ± 1.6	22.5 ± 0.8	−83.9 ± 1.5	−50.8 ± 4.5
L3	15.4 ± 1.5	15.8 ± 2.0	22.4 ± 1.4	−82.8 ± 3.2	−49.9 ± 2.4
L4	14.4 ± 1.4	15.6 ± 1.3	23.3 ± 1.6	−84.6 ± 1.6	−49.4 ± 3.4
L5	14.9 ± 1.3	14.3 ± 1.2	26.0 ± 3.1	−81.5 ± 2.3	−49.0 ± 5.4
L6	14.6 ± 1.6	14.4 ± 0.9	26.6 ± 1.2	−83.9 ± 2.2	−50.1 ± 4.0
S1	14.5 ± 1.7	12.9 ± 1.1	22.0 ± 1.6	−78.8 ± 2.9	−43.3 ± 2.1
S2	15.6 ± 1.9	13.1 ± 1.1	21.5 ± 2.4	−80.5 ± 3.7	−46.5 ± 4.6
S3	13.3 ± 1.0	12.3 ± 0.5	22.8 ± 1.0	−86.4 ± 2.6	−53.6 ± 3.6
S4	14.5 ± 1.4	12.2 ± 0.8	19.7 ± 1.0	−86.2 ± 4.4	−51.4 ± 4.0

The interfacet width (IFW) was greatest in the cervical and lumbar region with a maximum value of 26.6 mm at L6 and least in the thoracic region with a minimum of 18.4 mm at T8.

The articular facets from C4 to T1 were oriented dorsomedially (Table 5). The card angle around the y‐axis (CAY) measured between −62.7° (C4) and −72.1° (T1) and the card angle around the z‐axis (CAZ) was between −23° (C4) and −41.8° (T1). From T2 to T11 the orientation of the articular facets was changing in a dorsolateral direction. While remaining negative values of CAY from T2 to T11 from −53.5° (T11) and −70.8° (T4), the CAZ values were positive from 34° (T6) to 42.3° (T4). From T12 the orientation of the articular facets changed again in a dorsomedial direction with a steeper CAY from −75.5° (T12) to −86.4° (S3) and again with negative values of CAZ from −49° (L5) to −59.5° (T13).

#### 3.4.2 Intervertebral discs

Anterior disc height (IDH_a_) in the cervical region remained relatively constant between 4.7 mm and 5.8 mm (Table 6). Lowest values were measured in the middle thoracic region with a mean of 2.9 mm in T5/T6 and T6/T7. In the lumbar and sacral region, it increased again and the maximum height was measured with 15.5 mm in L6/S1.

**Table 6:** Anterior intervertebral disc height (IDH_a_) of the kangaroo (n = 8; mean ± SD in mm)

Disc	IDHa
C4/C5	4.7 ± 0.5
C5/C6	5.1 ± 1.1
C6/C7	5.1 ± 0.6
C7/T1	5.8 ± 0.7
T1/T2	4.4 ± 0.7
T2/T3	3.4 ± 0.4
T3/T4	3.9 ± 0.8
T4/T5	3.7 ± 1.1
T5/T6	2.9 ± 0.5
T6/T7	2.9 ± 0.4
T7/T8	3.2 ± 0.5
T8/T9	3.6 ± 0.6
T9/T10	3.9 ± 0.6
T10/T11	4.8 ± 0.4
T11/T12	6.0 ± 0.9
T12/T13	7.1 ± 0.4
T13/L1	7.7 ± 0.4
L1/L2	8.3 ± 0.9
L2/L3	8.3 ± 1.1
L3/L4	8.6 ± 1.0
L4/L5	9.0 ± 0.9
L5/L6	10.1 ± 0.9
L6/Sac	15.5 ± 1.6
Sac/S1	8.4 ± 1.1
S1/S2	9.5 ± 0.8
S2/S3	9.8 ± 0.7
S3/S4	10.9 ± 1.0

## 4 DISCUSSION

This is the first study, which provides anatomical data of the kangaroo spine (C4‐S4) and presents a quantitative comparison of kangaroo and human spinal anatomical structures, although anatomical differences cannot be completely captured by linear and angular measures.

For comparison with the human vertebral anatomy, quantitative data of C4 to L5 were taken from the works of Panjabi et al., since this is the most extensive collection of human anatomical data (Panjabi et al., 1991a; 1991b; 1992; 1993). Data for the human intervertebral disc were taken from Lu et al. (1999) for the cervical region; from Kunkel et al. (2011) for the thoracic region; and from Amonoo‐Kuofi et al. (1991) for the lumbar region.

**Figure 3:**
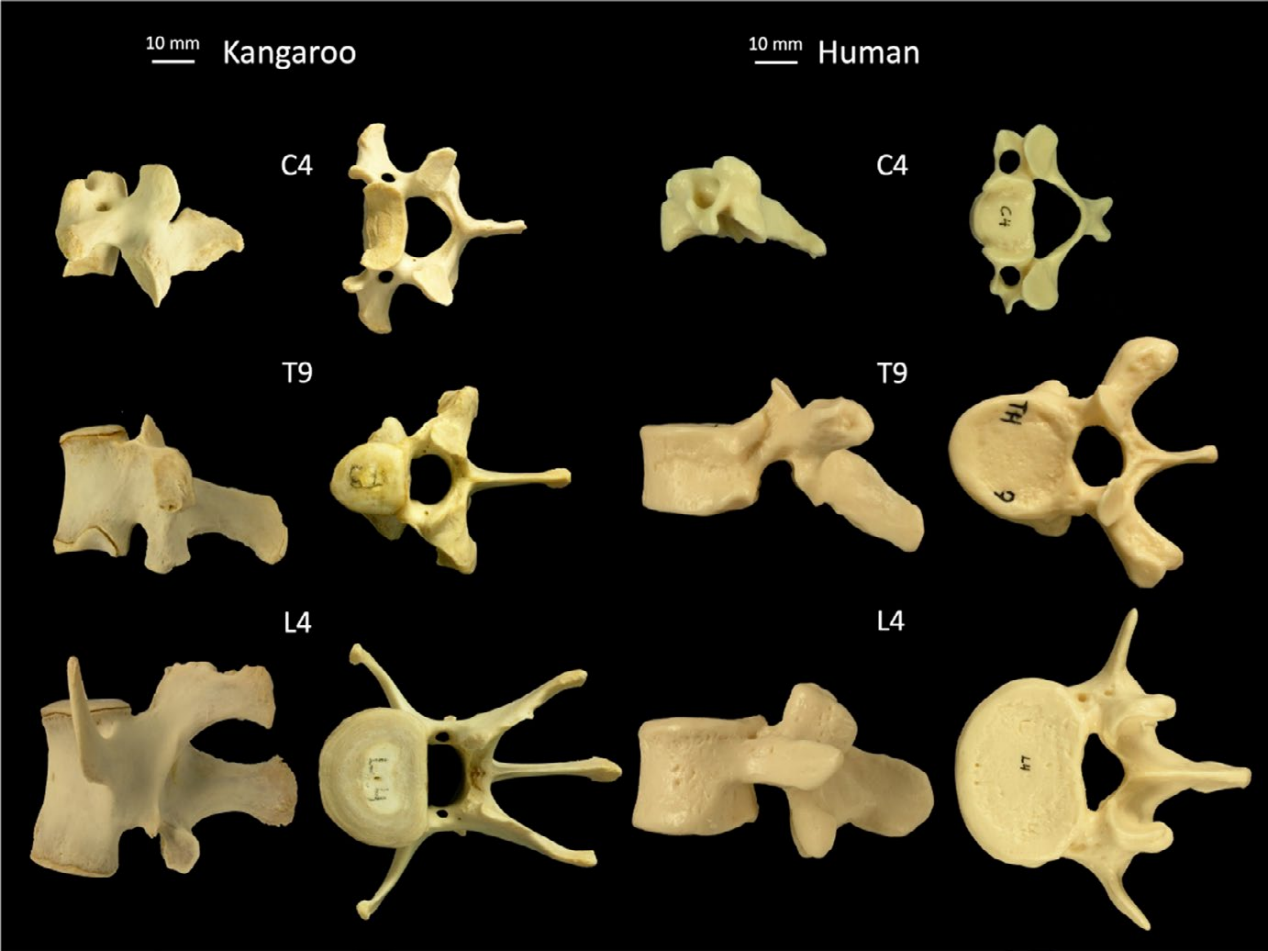
Sagittal and top view of C4, T9, and L4 vertebrae from kangaroo and human

From a first glance, the most similarities between the two species can be found for the cervical vertebra and for the vertebral bodies in the lumbar spine, at least in the transversal plane, but they are smaller in size and tend to be higher for the lumbar kangaroo spine (Figure 3). The most evident differences can be found comparing the different spinous processes, which represent the muscle attachments. We did not compare the dimensions of muscles, but it might be speculated that they are larger compared to the human spine. Although both species are somehow upright, these differences may be explained by the way how they move. Jumping probably requires more muscle strength than walking on two legs.

### 4.1 Vertebral bodies

**Figure 4:**
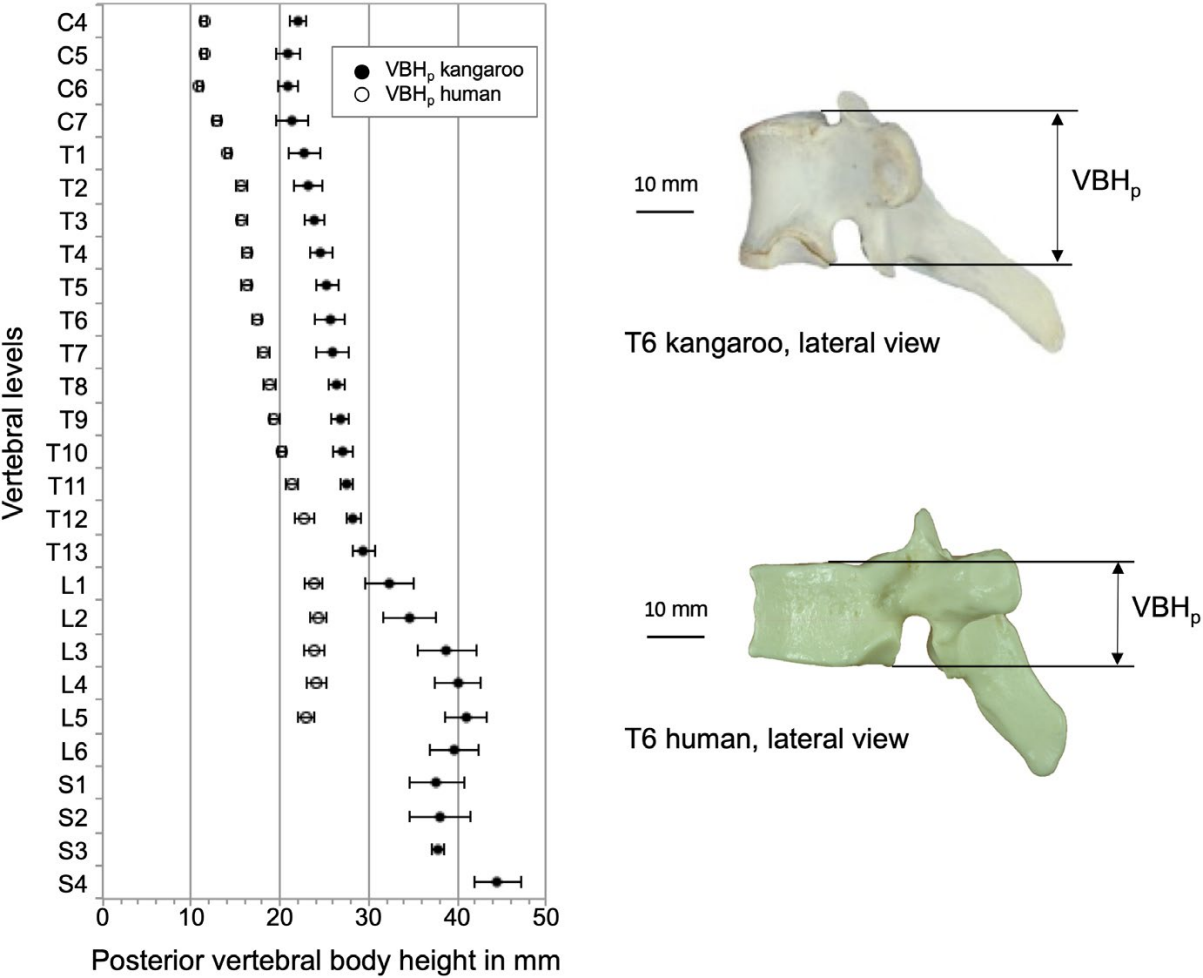
Values (mean ±SD) of posterior vertebral body height (VBH_p_) of the kangaroo spine from C4 to S4 in comparison with reported data for the human spine from C4 to L5 (Panjabi et al., 1991, 1992)

**Figure 5:**
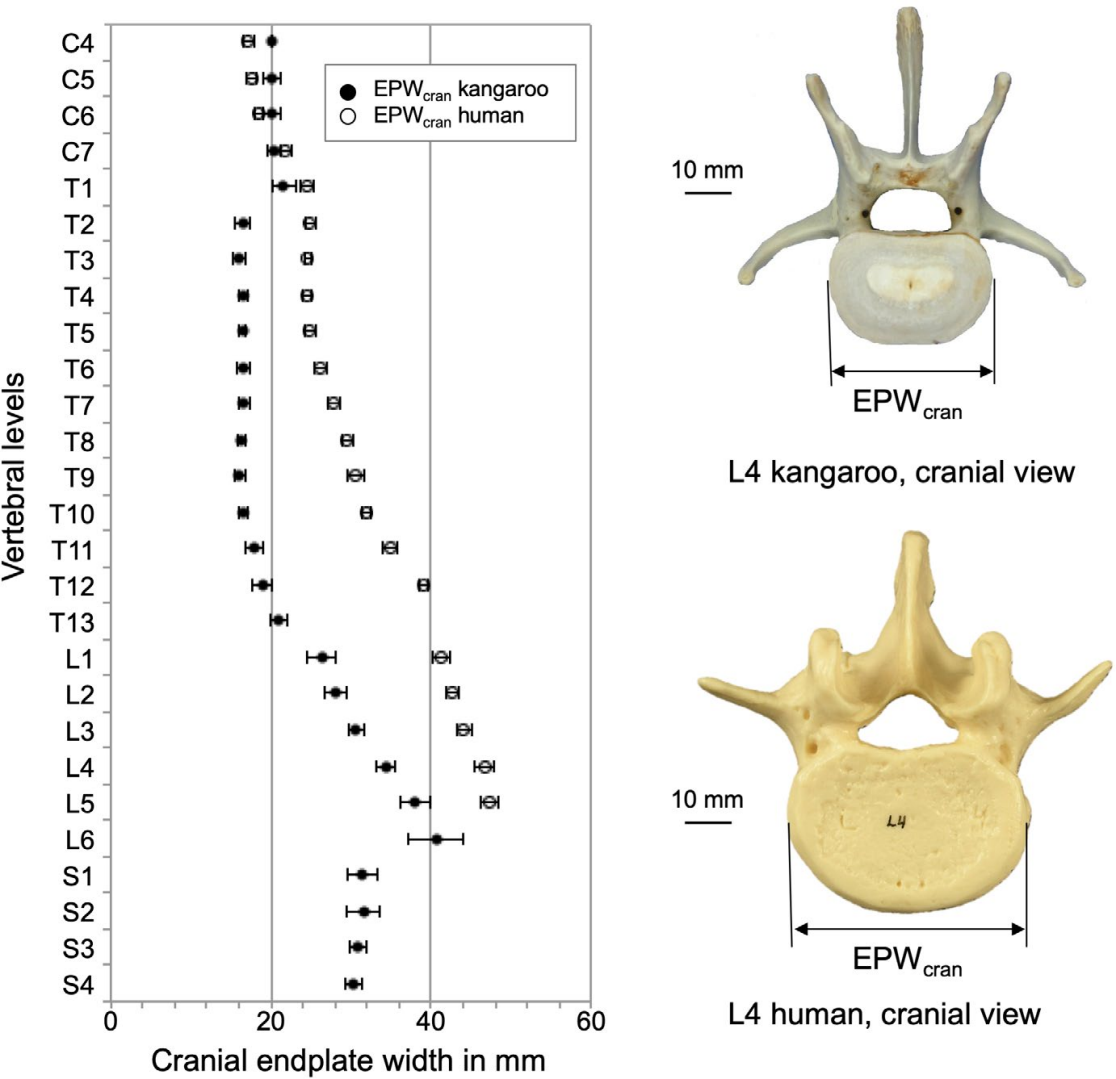
Values (mean ±SD) of cranial endplate width (EPW_cran_) of the kangaroo spine from C4 to S4 in comparison with reported data for the human spine from C4 to L5 (Panjabi et al., 1991, 1992)

**Figure 6:**
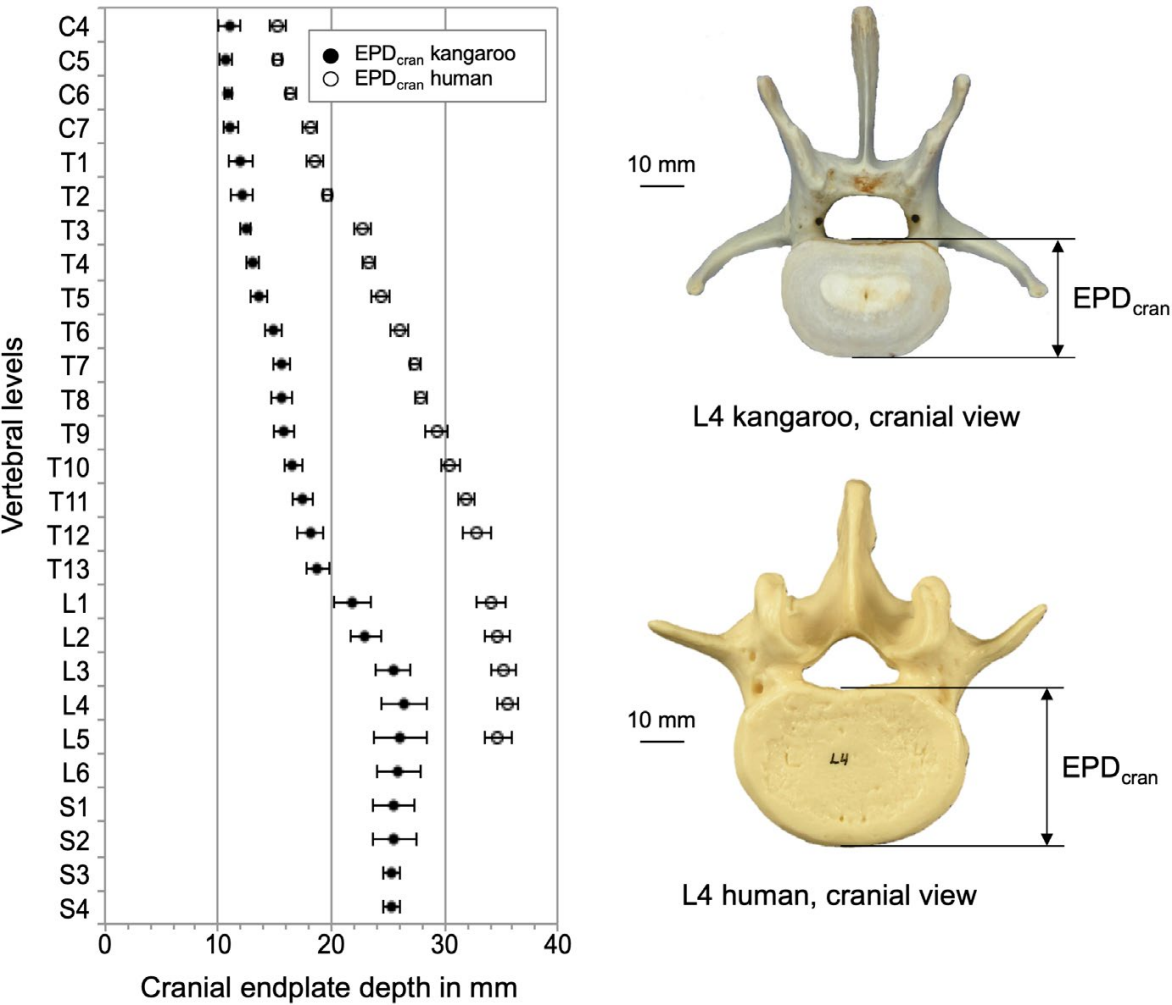
Values (mean ±SD) of cranial endplate depth (EPD_cran_) of the kangaroo spine from C4 to S4 in comparison with reported data for the human spine from C4 to L5 (Panjabi et al., 1991, 1992)

Kangaroo vertebral bodies were higher than the human ones along the entire spine (Figure 4). A difference between the kangaroo and the human vertebral bodies was that the kangaroo vertebras tend to be higher (VBH_P_) than broad (EPW_cran_), whereas generally the human vertebra was wider than tall (Figures 4 and 5). However, kangaroo and human had small cervical vertebrae, which allow great mobility of the head. Concerning EPD_cran_, the kangaroo vertebral bodies are less deep than the human ones (Figure 6). In general, the complete cranial endplate surface was smaller in the kangaroo than the human ones in all spinal regions.

### 4.2 Pedicles

**Figure 7:**
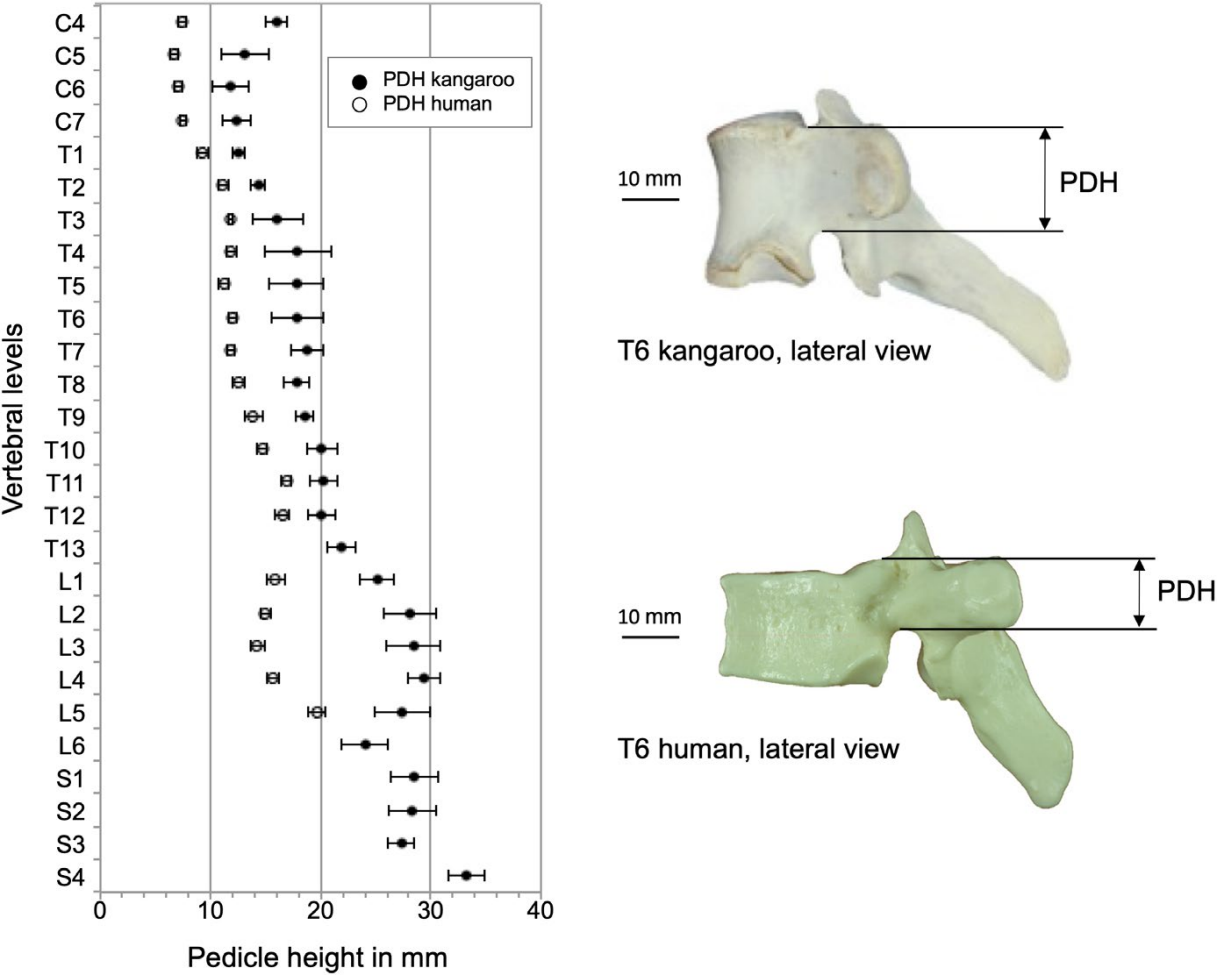
Values (mean ±SD) of pedicle height (PDH) of the kangaroo spine from C4 to S4 in comparison with reported data for the human spine from C4 to L5 (Panjabi et al., 1991, 1992)

**Figure 8:**
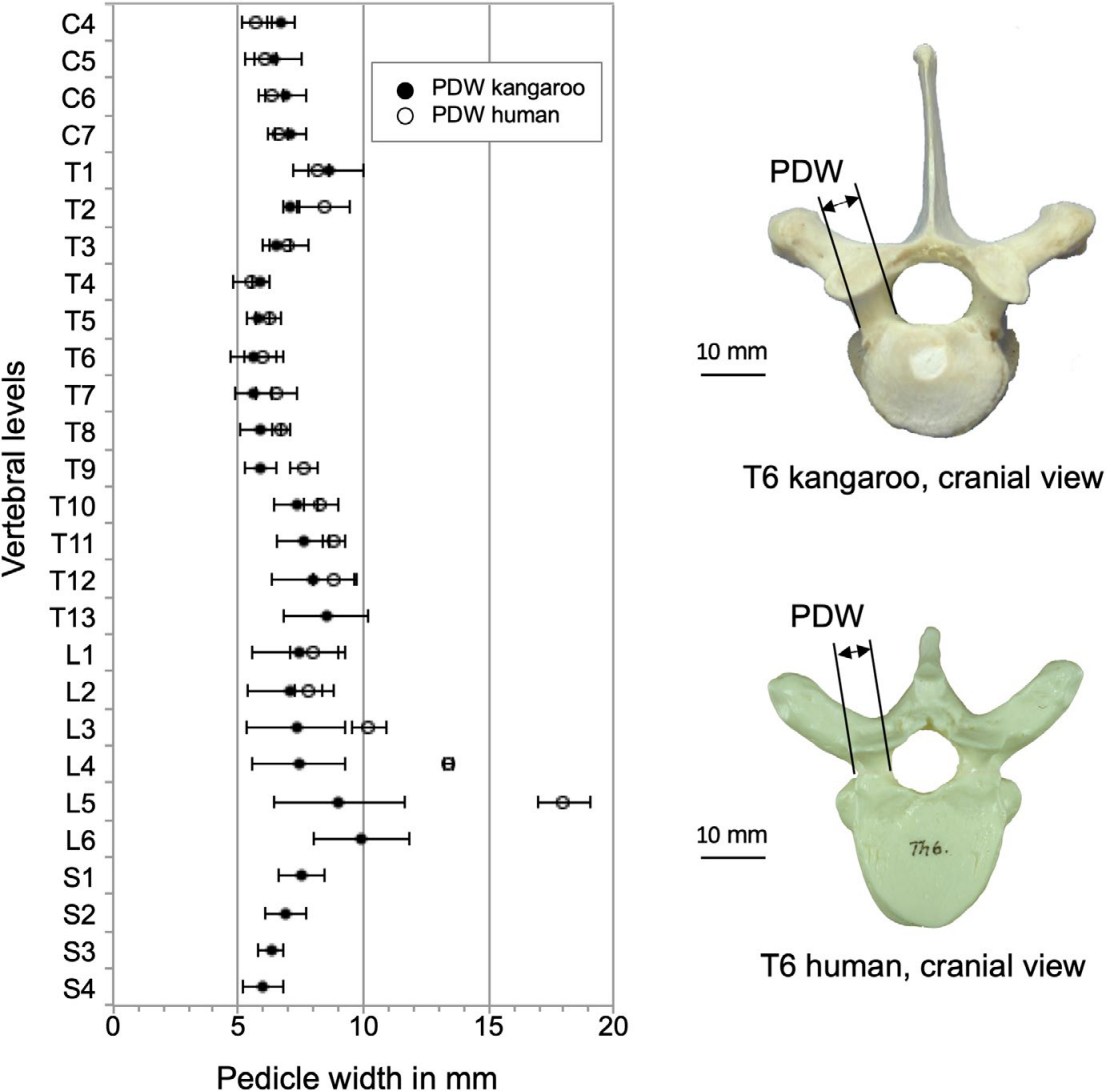
Values (mean ±SD) of pedicle width (PDW) of the kangaroo spine from C4 to S4 in comparison with reported data for the human spine from C4 to L5 (Panjabi et al., 1991, 1992)

The PDH of the kangaroo pedicles was always higher compared to the human ones for the entire kangaroo spine (Figure 7). However, a good correlation was found between the two species in PDW of the cervical and thoracic area (Figure 8). However, the human PDW was greater in the lower lumbar region.

### 4.3 Spinal canal

**Figure 9:**
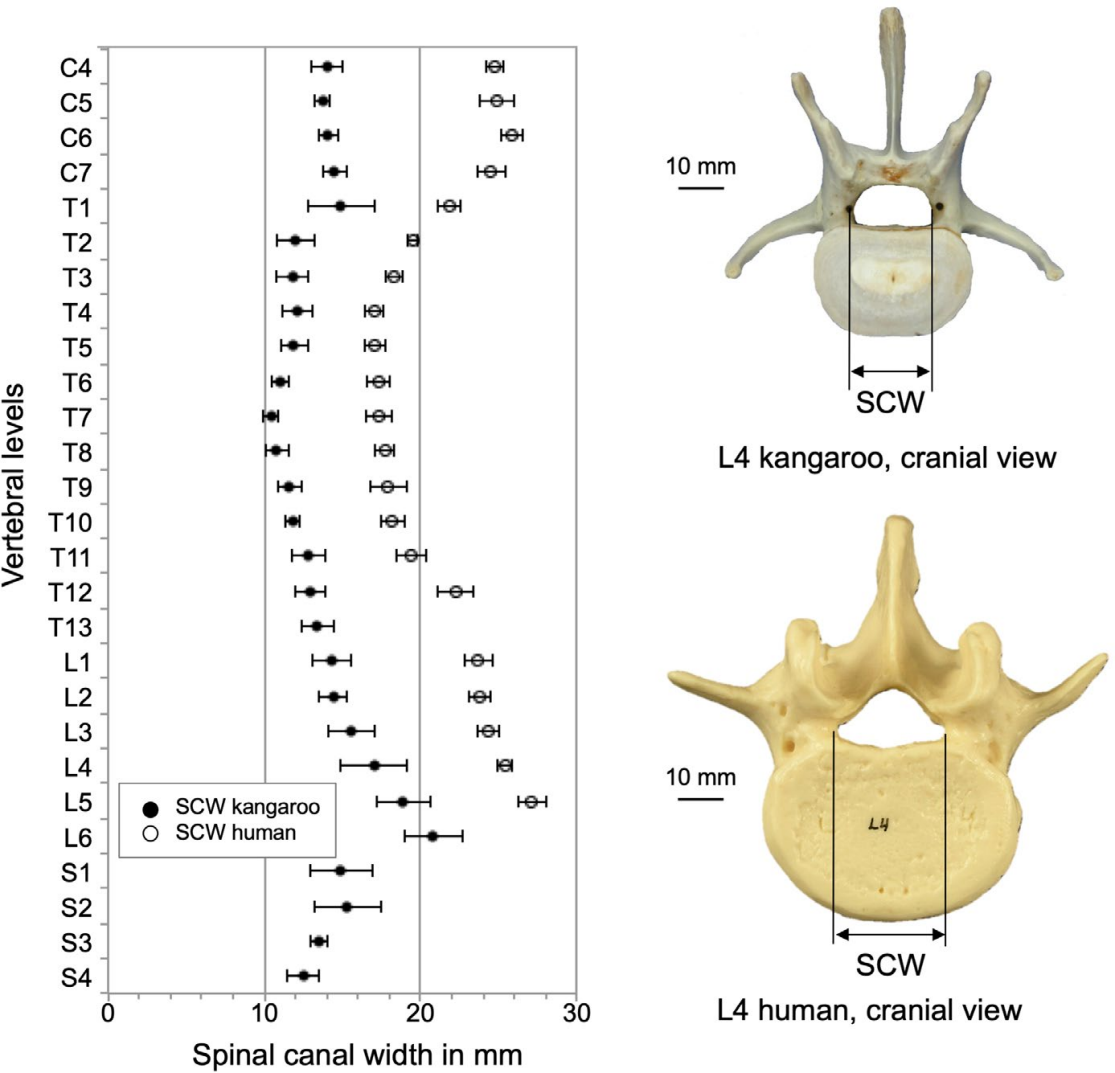
Values (mean ±SD) of spinal canal width (SCW) of the kangaroo spine from C4 to S4 in comparison with reported data for the human spine from C4 to L5 (Panjabi et al., 1991, 1992)

**Figure 10:**
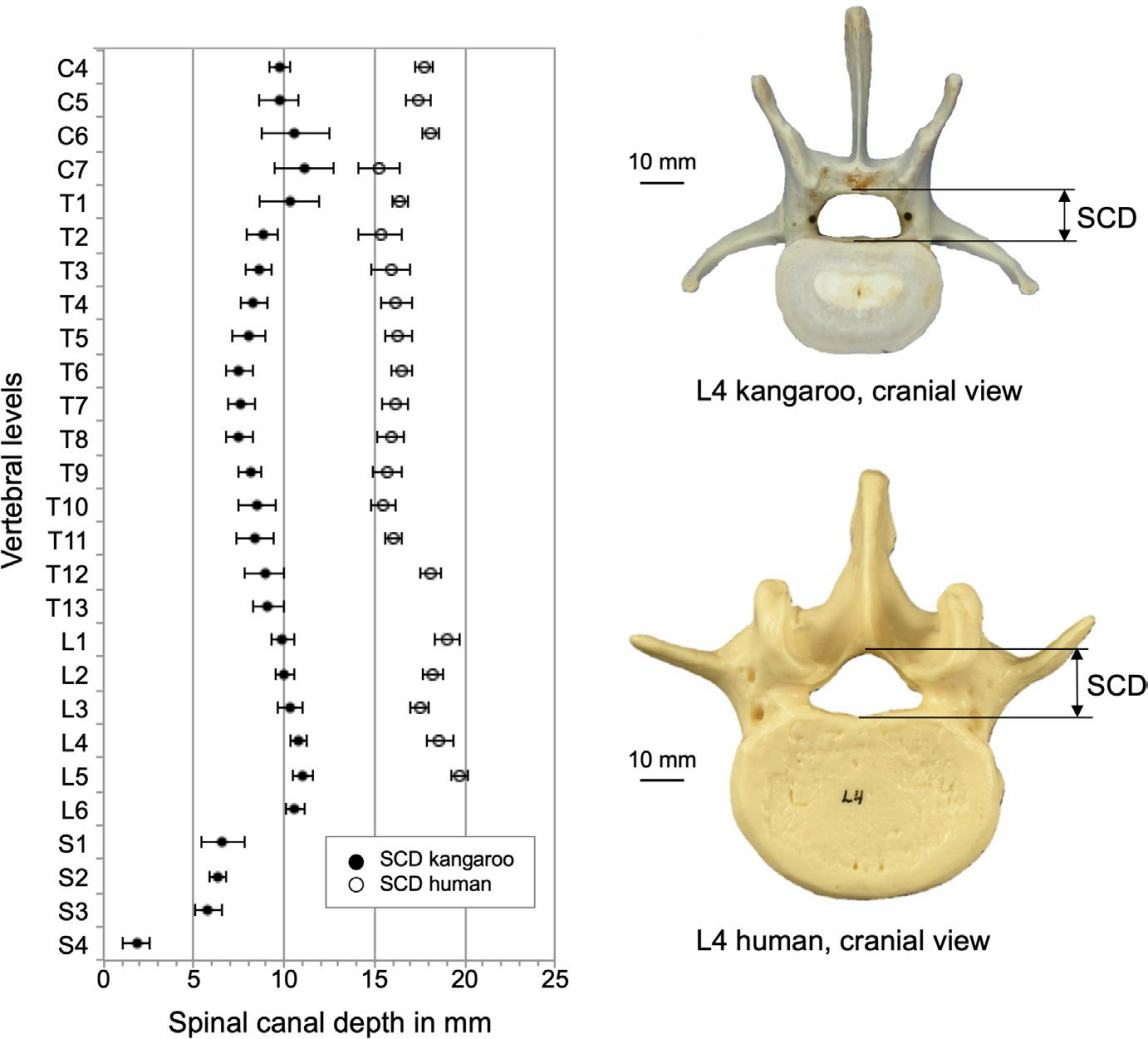
Values (mean ±SD) of spinal canal depth (SCD) of the kangaroo spine from C4 to S4 in comparison with reported data for the human spine from C4 to L5 (Panjabi et al., 1991, 1992)

The spinal canal from both species was a horizontal oval shape along the whole spine. Regarding SCW and SCD, the dimensions of the kangaroo spinal canal were always smaller than the human one (Figure 9 and Figure 10).

### 4.4 Spinous and transverse processes

**Figure 11:**
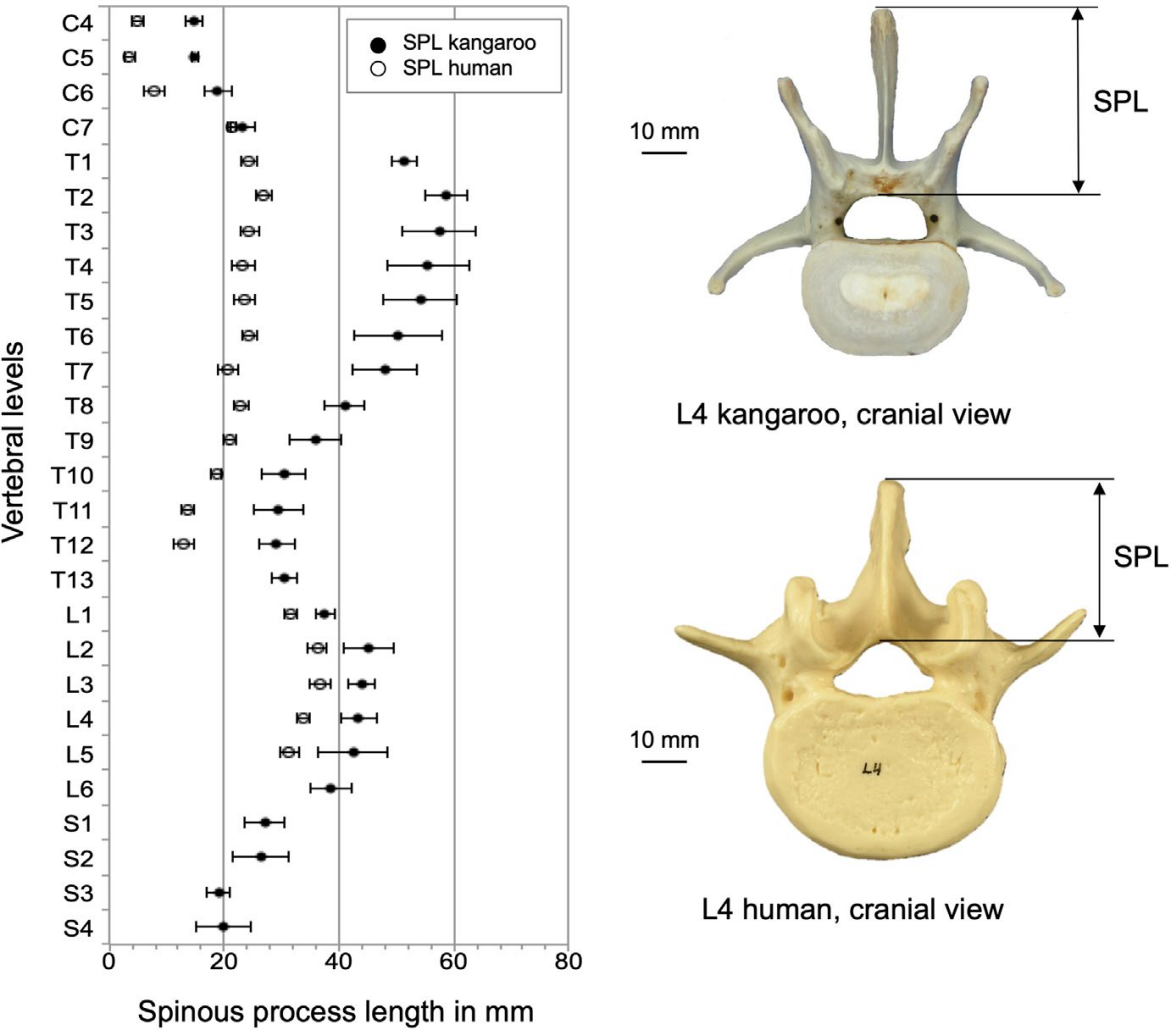
Values (mean ±SD) of spinous process length (SPL) of the kangaroo spine from C4 to S4 in comparison with reported data for the human spine from C4 to L5 (Panjabi et al., 1991, 1992). (Please note: The real length was measured and is reported, but the projection of the measuring length of SPL is shown in this figure.)

The spinous processes of the kangaroo are generally longer (SPL) than the human ones (Figure 11). In C7, the differences in SPL were lower, whereas in T1 the differences were observed to become substantially greater. In the lumbar region these differences in SPL get smaller again.

**Figure 12:**
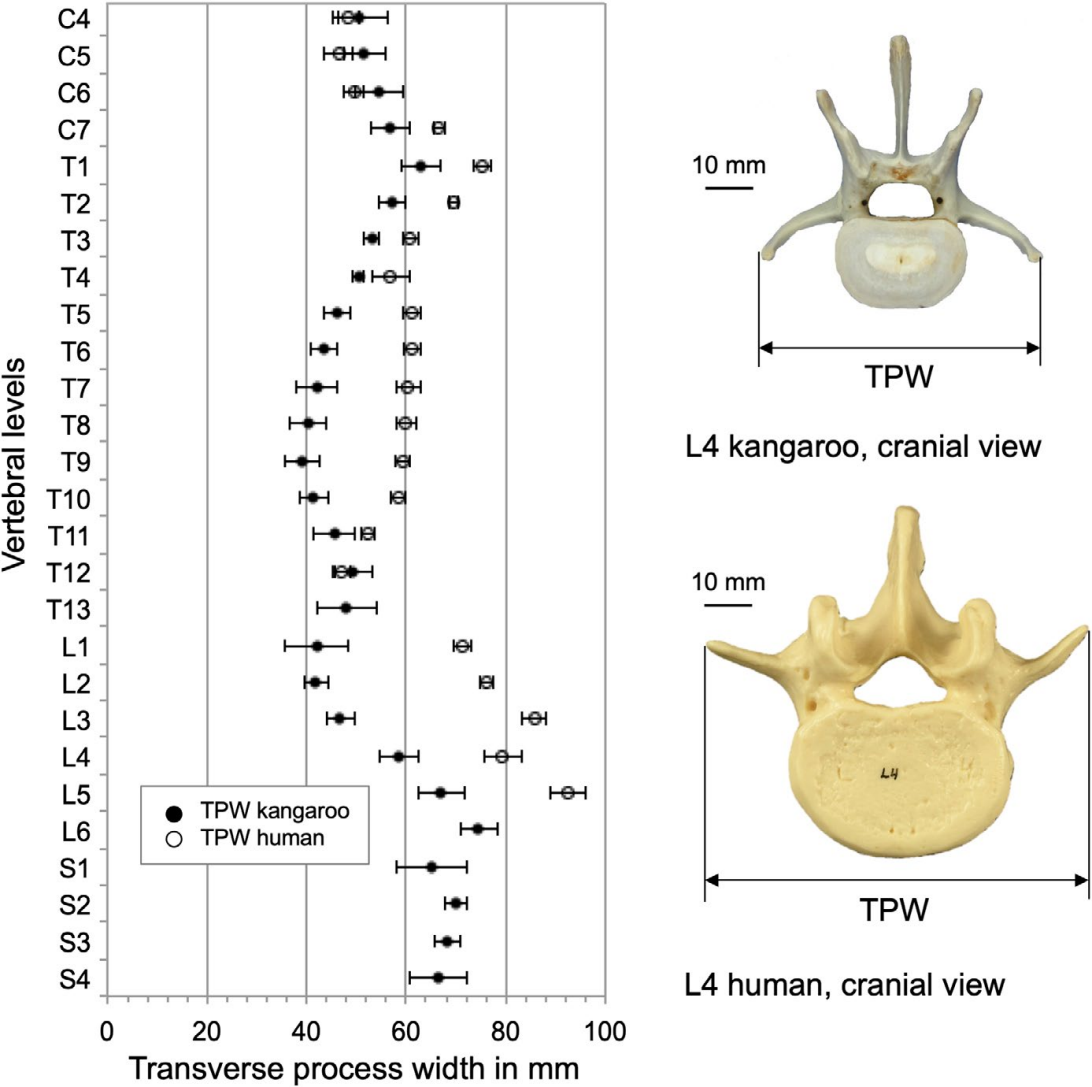
Values (mean ±SD) of transverse process width (TPW) of the kangaroo spine from C4 to S4 in comparison with reported data for the human spine from C4 to L5 (Panjabi et al., 1991, 1992)

The transverse process width (TPW) in the middle cervical region was fairly similar for both species (Figure 12). In the thoracic (with exception of T12), the human TPW is large and these differences further increased in the lumbar region.

### 4.5 Facet joints

**Figure 13:**
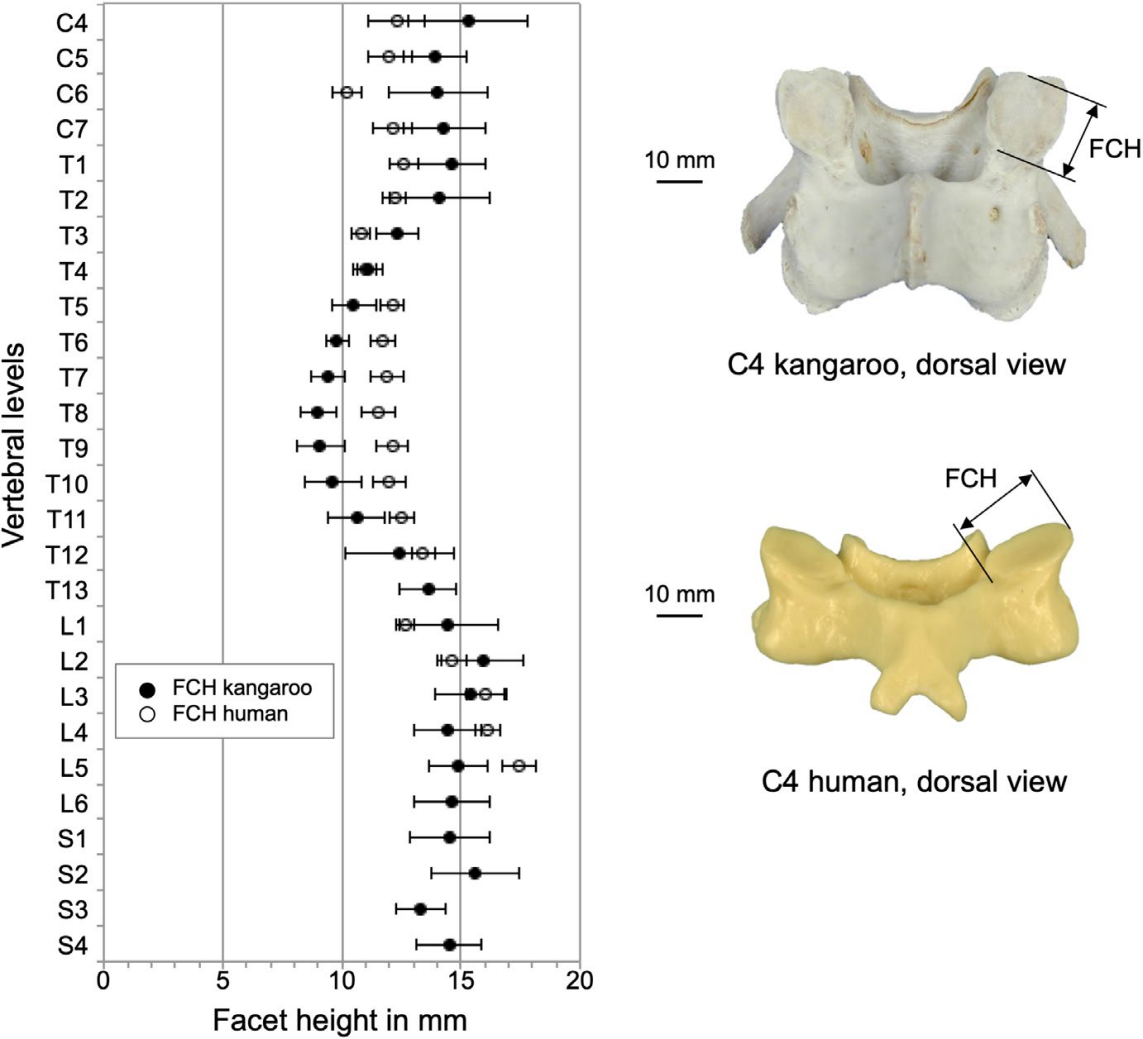
Values (mean ±SD) of facet height (FCH) of the kangaroo spine from C4 to S4 in comparison with reported data of the human spine from C4 to L5 (Panjabi et al., 1993)

**Figure 14:**
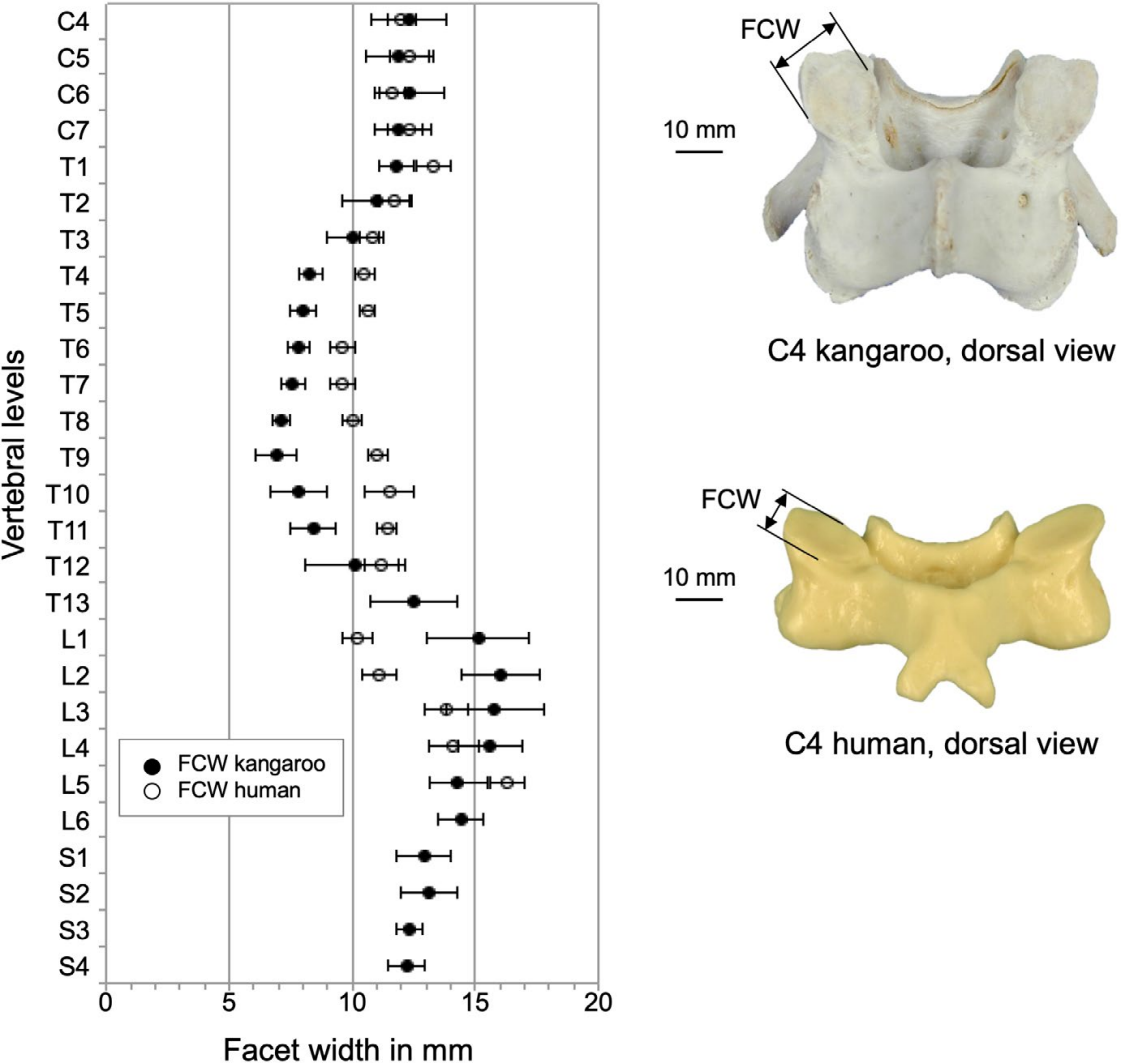
Values (mean ±SD) of facet width (FCW) of the kangaroo spine from C4 to S4 in comparison with reported data of the human spine from C4 to L5 (Panjabi et al., 1993)

**Figure 15:**
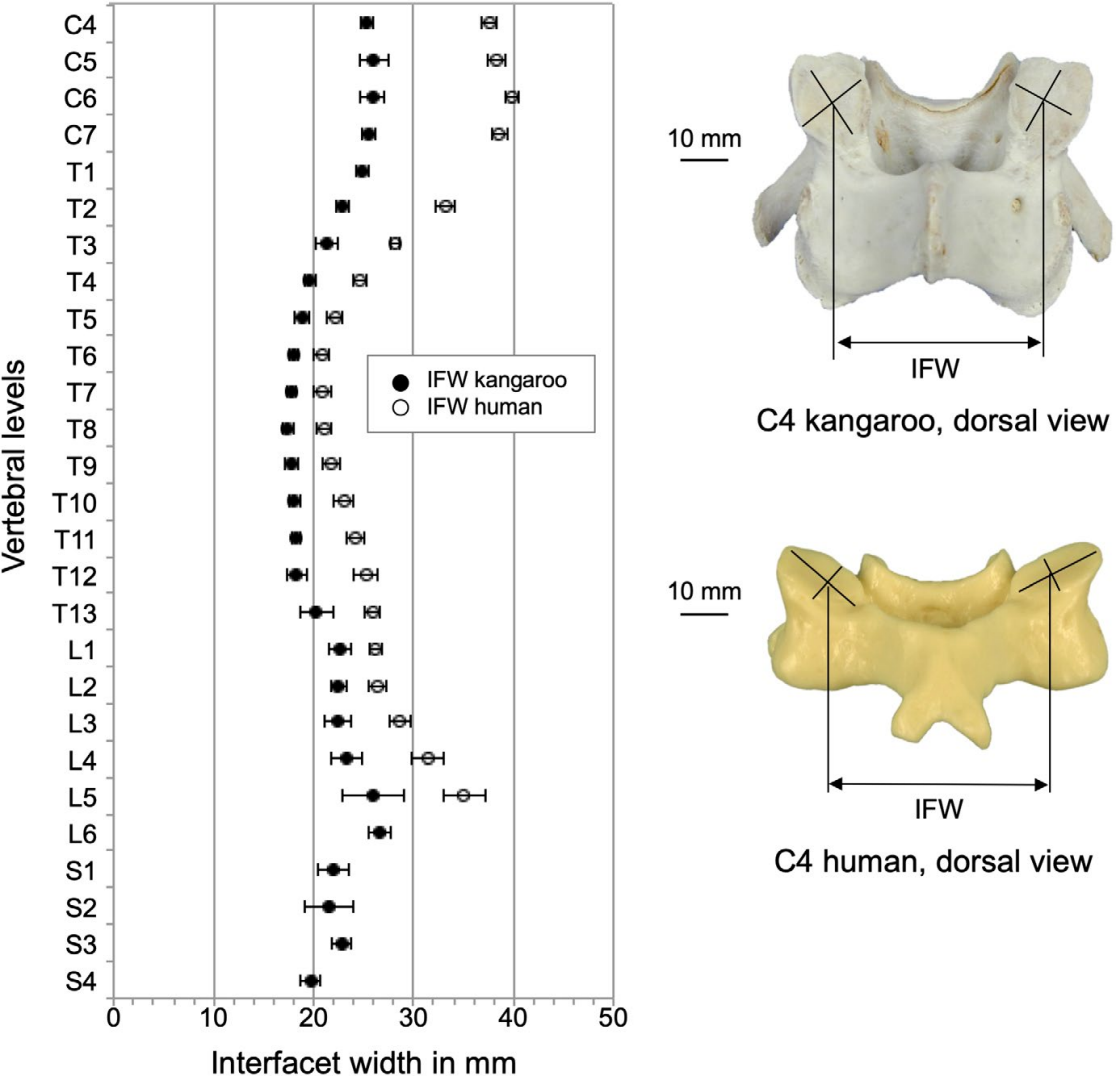
Values (mean ±SD) of interfacet width (IFW) of the kangaroo spine from C4 to S4 in comparison with reported data of the human spine from C4 to L5 (Panjabi et al., 1993)

Facet height (FCH) and facet width (FCW) were quite similar between kangaroo and human (Figure 13 and Figure 14). Small differences were observed between the two species in the thoracic and lumbar regions. Generally, the human IFW was greater than the kangaroo, but the progression was similar for both species (Figure 15). Major differences can be seen in the cervical region; in the middle thoracic region, they were lower.

**Figure 16:**
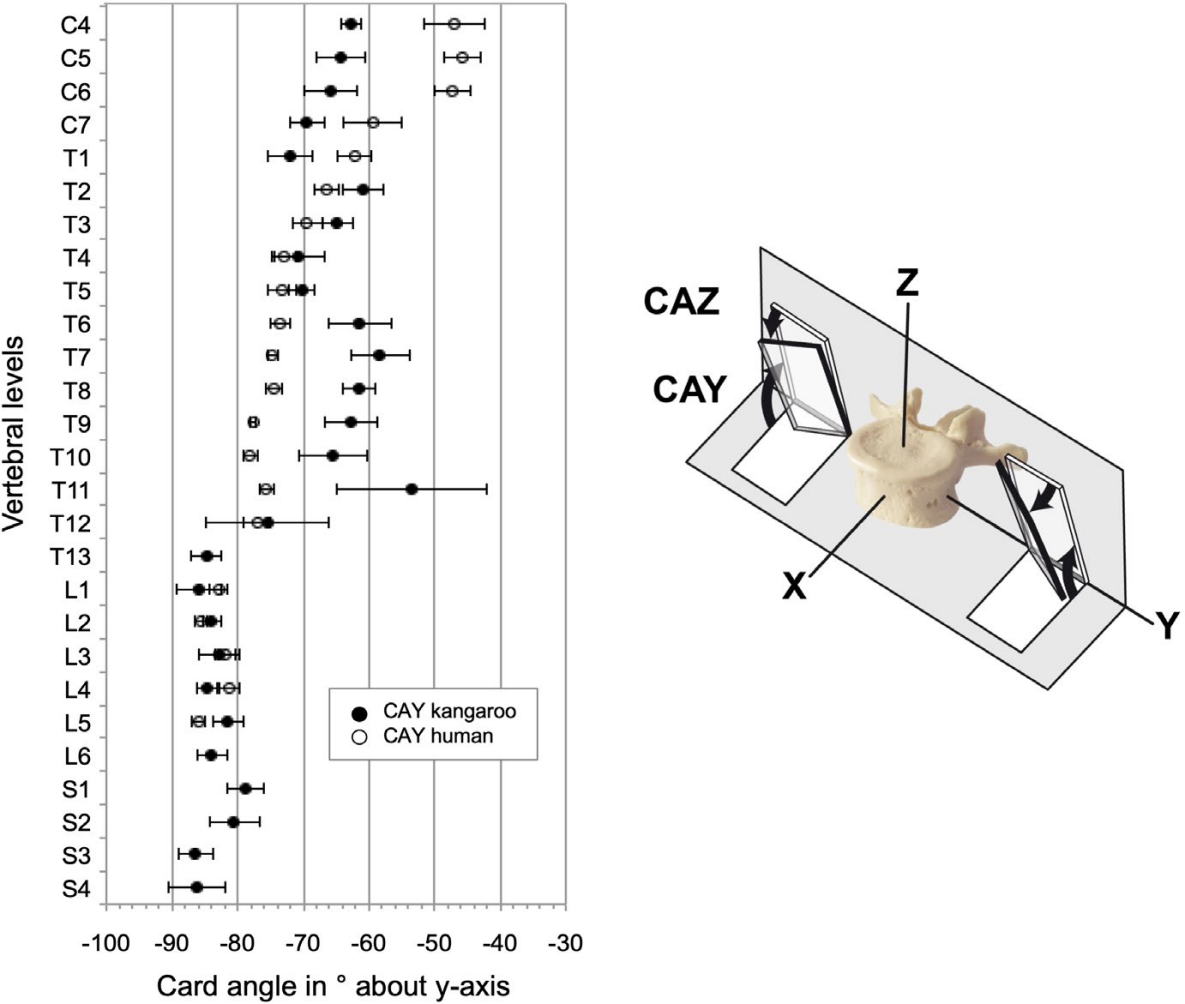
Values (mean ±SD) of facet plane orientation about the y‐axis (CAY) (adapted from Panjabi et al., 1993) of the kangaroo spine from C4 to S4 in comparison with reported data of the human spine from C4 to L5 (Panjabi et al., 1993)

**Figure 17:**
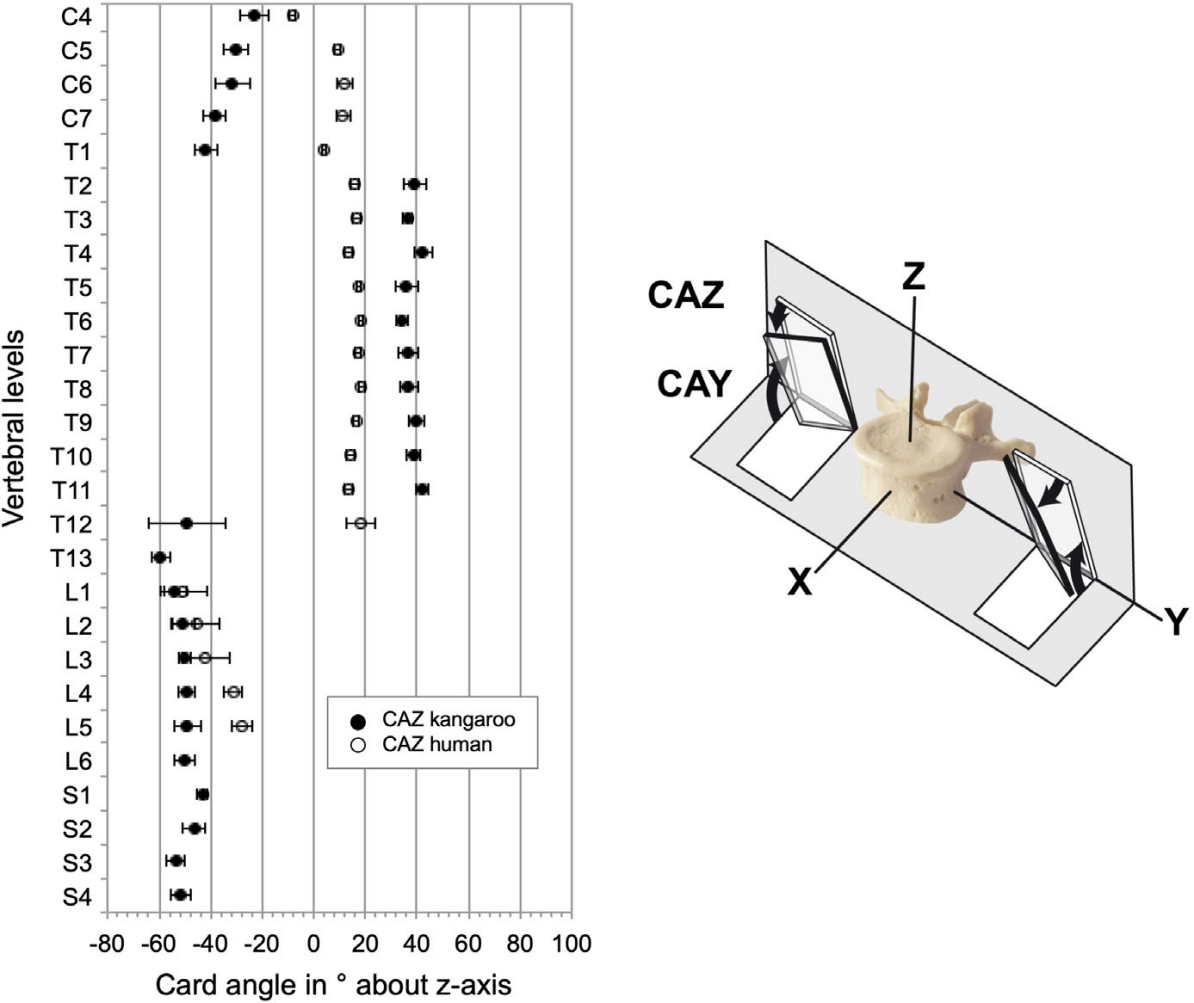
Values (mean ±SD) of facet plane orientation about the z‐axis (CAZ) (adapted from Panjabi et al., 1993) of the kangaroo spine from C4 to S4 in comparison with reported data of the human spine from C4 to L5 (Panjabi et al., 1993)

Regarding the orientation of the facet plane in the cervical region, the cranial articular facets of the kangaroo are facing dorsomedial and the orientation of the human ones is slightly dorsolateral (Figure 16 and Figure 17). In the thoracic spine, the angular position of the articular facets of the kangaroo is dorsolateral, the human facets are lying with CAZ of around 20° nearly in the coronal plane. In the lumbar region, the orientation of the facets from both species was even nearly identically. They stand almost vertically and point in dorsomedial direction. This can be understood as an adaptation to similar ventral shear forces and torsional stresses related to bipedal movement.

### 4.6 Intervertebral discs

**Figure 18:**
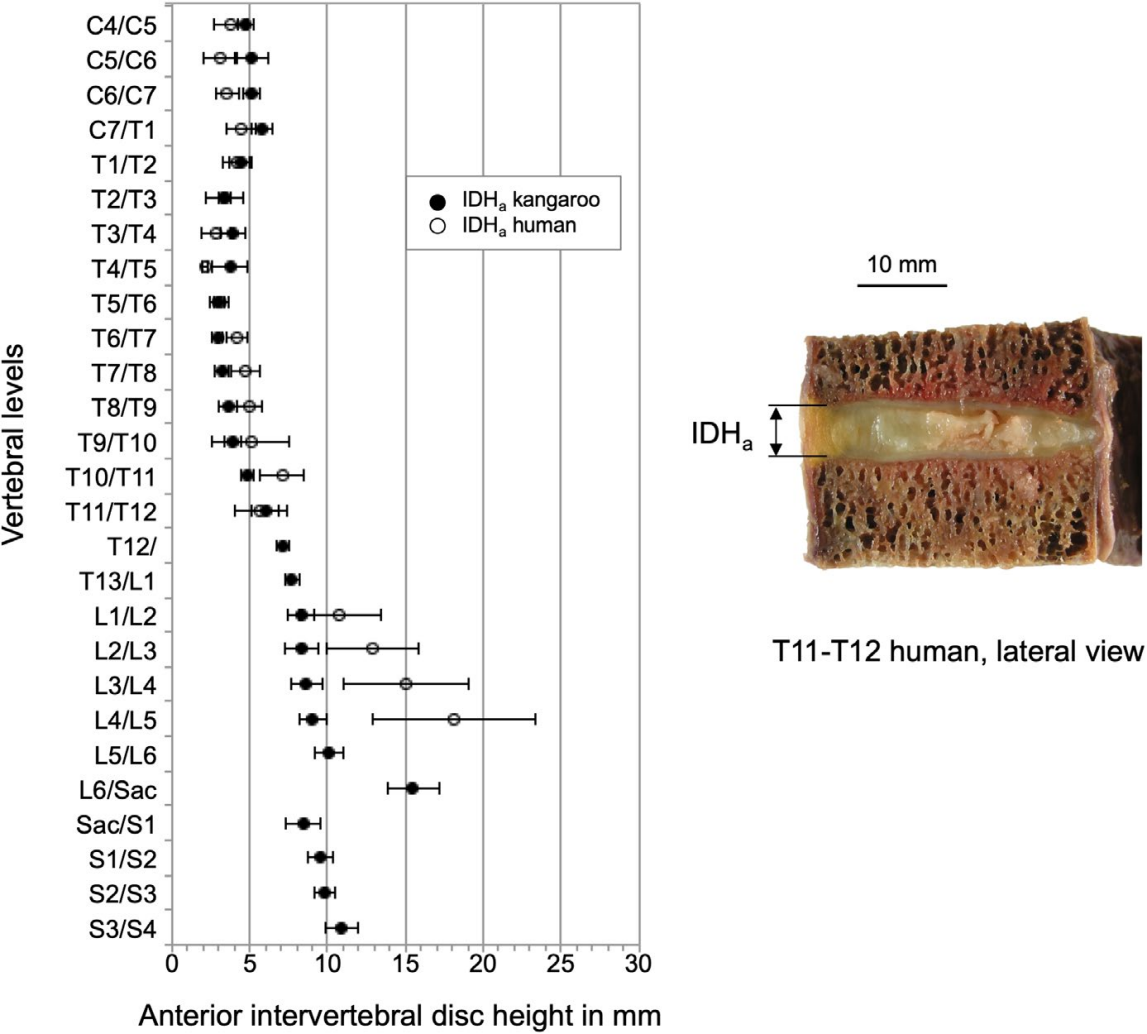
Values (mean ±SD) of anterior intervertebral disc height (IDH_a_) of the kangaroo spine from C4/C5 to S3/S4 in comparison with reported data of the human spine from C4/C5 to L4/L5 (Amonoo‐Kuofi, 1991; Kunkel et al., 2011; Lu et al., 1999)

The values of kangaroo and human IDH_a_ in the cervical and thoracic region, are very similar (Figure 18). In the lumbar spine the human IDH_a_ increases more than the IDH_a_ of the kangaroo.

## 5 CONCLUSION

In some area, like the cervical and thoracic pedicle width (PDW) or the anterior intervertebral disc height (IDH_a_) in the cervical and thoracic region, the anatomy shows a good correlation between kangaroo and human. For some measured parameters, like the pedicle height (PDH) in the cervical and thoracic region or the facets, minor variations occur. Stronger differences are evident for example in the length of the spinous process (SPL) of the thoracic region or in the lumbar pedicle height (PDH).

However, not only the morphometry determines the value of a model but also tissue material properties of bones, intervertebral discs, and ligaments are just as important. Some of these data are represented indirectly by load‐deformation characteristics, which can be determined with flexibility tests (Chamoli et al., 2014, 2015; Sabet et al., 2011).

To decide, whether the kangaroo spine is a suitable model, further studies should follow.

## AUTHOR CONTRIBUTIONS

Prof. Dr. Hans‐Joachim Wilke: Idea of the project and contributions to the concept/design; Interpretation of the data; Support writing the paper. Critical revision of the manuscript and approval of the article. PD Dr. Annette Kienle: Contributions to the concept/design. Critical revision of the manuscript and approval of the article. Dr. Volker Michael Betz: Acquisition of data. Data analysis and interpretation. Critical revision of the manuscript and approval of the article. Dipl.‐Ing. (FH) Karin Werner: Support with the figures and drafting of the manuscript.

## Data Availability

The data that support the findings of this study are available from the corresponding author upon reasonable request.
